# Assessing balance via cross-line laser in individuals with chronic ankle instability

**DOI:** 10.3389/fspor.2025.1688891

**Published:** 2025-11-14

**Authors:** Madison Swails, Emma Hardy, Jeanne Dury, Abbey C. Thomas, Shelley W. Linens, Luke Donovan

**Affiliations:** 1 Department of Applied Physiology, Health, and Clinical Sciences, University of North Carolina at Charlotte, Charlotte, NC, United States; 2 Laboratoire UR 4660-C3S Culture, Sport, Santé, Société, Université Marie et Louis PASTEUR, Besancon, France

**Keywords:** balance, chronic ankle instability, force plate, postural control, external feedback

## Abstract

**Introduction:**

Chronic ankle instability (CAI) is a condition characterized by recurring lateral ankle sprains and bouts of instability. Individuals with CAI have poor balance, a factor in the development, progression, and recovery of the condition. Individuals with improved balance report fewer CAI-related symptoms. Thus, accurate balance assessment is crucial to recovery from CAI. The gold standard for measuring balance is using force plates. Although sensitive to subtle changes in balance, the force plate's clinical utility is diminished due to high associated costs. Alternatively, a low-cost cross-line laser may serve as a surrogate to the gold standard within a clinical setting. Therefore, the purpose of this study was to validate the cross-line laser as a tool for balance assessment in comparison with the gold-standard force plate.

**Methods:**

Twenty-four individuals with CAI in a single-limb stance balanced on a force plate for three 10 s trials in eyes-open and eyes-closed conditions with a cross-line laser strapped to the top of their foot. A camera in front of the force plate captured the movement of the cross-line laser. Utilizing a tracking software, a virtual marker was placed on the cross-line laser that quantified the movement of the cross-line laser.

**Results:**

The results of this study found that cross-line laser outcomes, such as speed, horizontal velocity, total distance, and resultant velocity measures, had moderate-to-strong relationships to force plate outcomes, such as center of pressure (CoP) average velocity, and CoP 95% area (*r* = 0.46–0.87) and CAI patient-reported symptoms (*r* = 0.44–0.52) indicating that these measures could be used twofold.

**Conclusion:**

This study validates the cross-line laser as a balance assessment tool that may serve as a low-cost instrument to quantify balance.

## Introduction

Lateral ankle sprains (LASs) are the most reported musculoskeletal injury among physically active individuals (1, 2). Unfortunately, dependent on activity type, approximately 20%–75% of individuals who sustain a LAS develop a condition characterized by frequent bouts of instability and/or repetitive LAS known as chronic ankle instability (CAI) (1–3). Consequences of CAI include diminished health-reported quality of life (4, 5), decreased physical activity (6), and increased risk of developing ankle osteoarthritis (OA) (7–9). The definitive cause of CAI remains unknown; however, balance has been identified as a key factor regarding the development, progression, and recovery of CAI (10, 11). In this context, balance is operationally defined as the ability to maintain one's center of mass within their respective base of support (12).

As such, accurately assessing balance in a clinical setting is imperative to identifying individuals at risk of LAS/CAI and for monitoring patient progress as an individual recovers from the condition (13).

Presently, force plates (14–17) are considered the gold standard for assessing balance via measures of center of pressure (CoP). Among individuals with CAI, force plates have been shown to be capable of detecting differences in CoP 95% area and average velocity when compared with healthy individuals (18). Force plates are also sensitive enough to detect improvements in these measures following balance training in individuals with CAI (14, 19). Despite the associated precision, the utility of force plates in most clinical settings may be diminished due to their high costs (often >$5,000). On the contrary, numerous non-instrumented and clinically feasible balance tests have been developed, such as the Balance Error Scoring System (BESS) (17, 20, 21), Star Excursion Balance Test (SEBT) (19), and foot-lift test (17, 21). A previous review (17) has shown that these non-instrumented balance tests can identify differences in balance between individuals with and without CAI. Although these tests have clinical value, due to the somewhat subjective nature of the scoring criteria, the ability of these tests to capture incremental changes in balance (changes one may expect during rehabilitation progression) remains somewhat in question (22–27). Therefore, a low-cost objective instrumented balance assessment tool is necessary.

One potential solution is a cross-line laser. In theory, the laser light projection on a wall would suggest a corresponding change in the position of the laser pointer. Stated differently, if a laser pointer were shone onto a wall, the position of the laser would not change unless the position of the laser pointer changed. This notion was previously tested, where a cross-line laser was shown capable of estimating plantar pressure during treadmill walking among individuals with CAI (28, 29). Furthermore, other studies have shown that when using a cross-line laser as a cue during balance and walking tasks, individuals with CAI can alter their biomechanical profile by manipulating the position of the laser (28, 30). Therefore, the overall purpose of this study is to validate a cross-line laser against gold-standard force plate measures as a means to assess balance among individuals with CAI. Specifically, we aimed to determine the relationship between variables derived from the movement of the cross-line laser (vertical velocity, horizontal velocity, resultant velocity, speed, total distance) with commonly reported variables from the force plate (CoP 95% area and CoP average velocity) during single-limb eyes-open and eyes-closed balance conditions. To establish the clinical implications of using a cross-line laser to measure balance, we secondarily aimed to determine the relationship between cross-line laser measures and participant-reported CAI symptoms.

## Materials and methods

### Participants

Twenty-four participants (21.0 ± 3.0 years, 170.0 ± 8.6 cm, 75.4 ± 14.8 kg, 10 male, 14 female) with self-reported CAI recruited from a university setting were included in this descriptive laboratory study. Individuals were eligible to enroll in this study if they engaged in at minimum light physical activity (brisk walk for 30 min at least three times per week) and were classified as having CAI in accordance with the selection criteria developed by the International Ankle Consortium (IAC) (31). Sample size selection was based on previously published literature that utilized the cross-line laser tool among participants with CAI (28–30).

To be considered having CAI, all participants reported (31):

1. A history of at least one significant ankle sprain occurring >12 months prior to the start of the study
2. At least two episodes of giving way in the previous 6 months
3. Self-reported ankle instability defined by scoring >10 on the Identification of functional ankle instability (IdFAI) questionnaire

Participants were not eligible for the study if they reported a history of lower extremity injury within the past 3 months and a history of surgery or fracture to the involved limb (31). In the event that the patient had experienced bilateral ankle sprains, they were instructed to choose which ankle was perceived to have worse symptoms and then complete the questionnaires regarding that limb.

### Instrumentation

**Figure 1:**
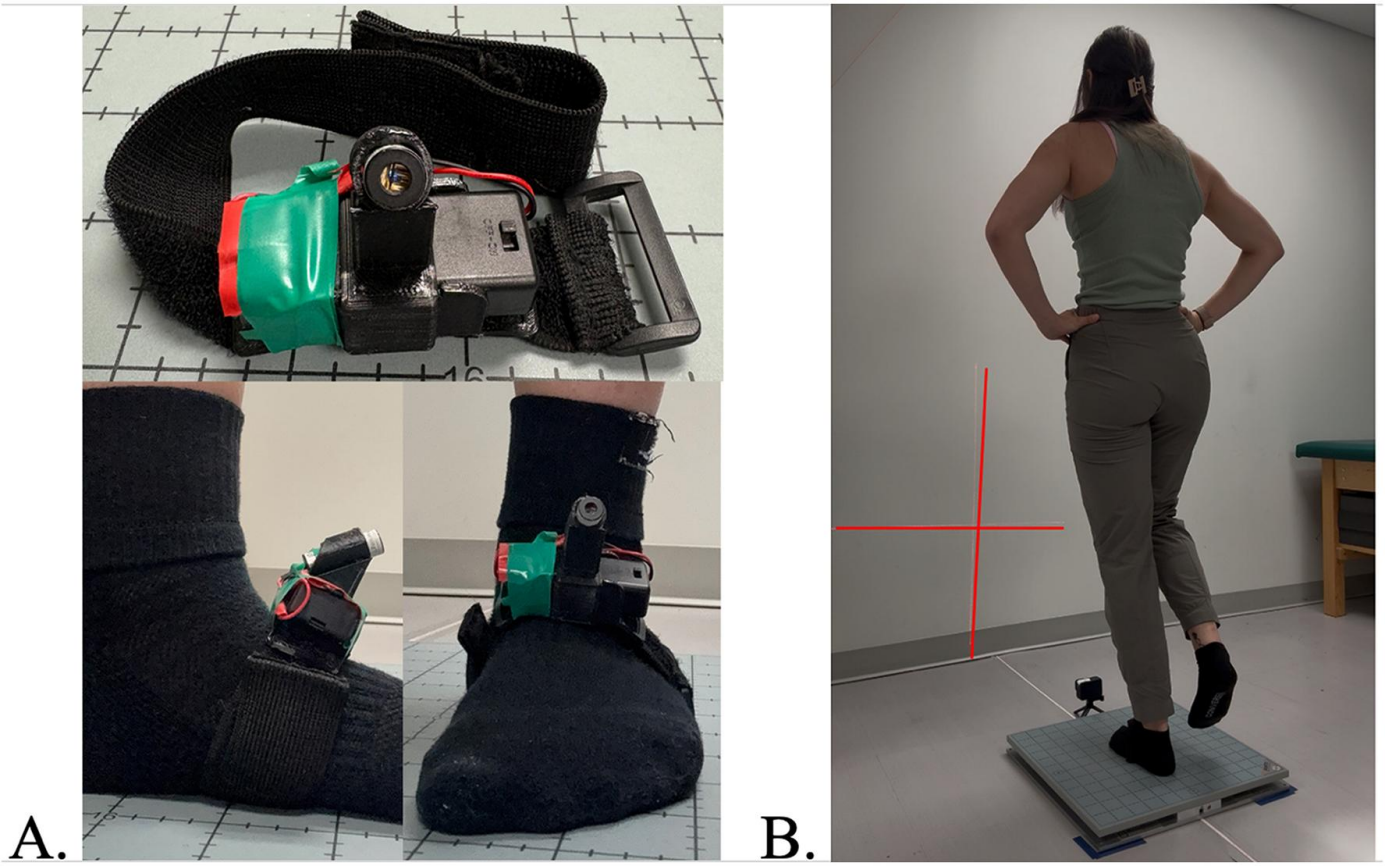
**(A)** Cross-line laser with a custom mount and a strap that was placed on the dorsal surface of the participant’s foot during single-limb stance. **(B)** Single-limb balance stance position was maintained for the entire 10 s trial while standing on a force plate and a cross-line laser strapped to the dorsal surface of their foot. A camera in front of the force plate captures the movement of the cross-line laser.

Participant self-reported ankle function was quantified using the Foot and Ankle Ability Measure (FAAM) Activities of Daily Living (ADL) and Sport subscale questionnaires (32). Furthermore, self-reported physical activity was measured by way of the International Physical Activity Questionnaire Short Form (IPAQ-SF) (33). The gold-standard measures of single-limb balance were assessed using a force plate (AccuSway Optimized, Advanced Mechanical Technology, Inc.; Watertown, MA, USA, 2023) and the corresponding software (Balance Clinic, AccuSway Optimized, Advanced Mechanical Technology, Inc.; Watertown, MA, USA). Force plate data were sampled at a 60 Hz frequency and processed with a fourth-order, zero-lag, low-pass Butterworth filter with a cutoff frequency of 5 Hz (19). The experimental cross-line laser tool was comprised of a class IIIA laser diode (Calpac Lasers; Steamboat Springs, CO, USA), battery holder, two AA batteries, and a custom-made mount with a strap (Figure 1A). The laser output during the single-limb balance trials was recorded using a video camera (GoPro Inc., HERO12 Black; San Mateo, CA, USA) recording at a sampling rate of 60 frames per second, which matched the force plate frequency. The camera was controlled using the manufacturer's remote (the remote for HERO12 Black; San Mateo, CA, USA). The video recordings of the laser movement were processed within a free virtual tracking software (Kinovea, Version 0.9.5V2; Free open source software).

### Procedures

Participants first reported to a biomechanics laboratory on the university's campus. Prior to testing, all participants completed the informed consent process (IRB 22-0954). Once participants provided informed consent, they completed the questionnaire required to confirm the presence of CAI (IdFAI), as well as descriptive questionnaires to quantify perceived ankle function (FAAM-ADL and FAAM-Sport) and physical activity (IPAQ-SF).

Utilizing commonly reported single-limb balance assessment procedures (19), participants completed three 10 s trials of single-limb balance in both an eyes-open and eyes-closed condition. Prior to the start of the balance trials, participants had the cross-line laser fixed to the dorsal aspect of their limb classified as having CAI (Figure 1A). Once the laser was mounted to the limb with CAI, participants positioned their foot in the center of the force plate. Next, the laser was positioned on the mount in a manner so that the output would project onto the wall with the vertical axis running parallel to the wall while the horizontal axis was parallel to the floor. Considering the natural angulation of the foot dorsum and to ensure that the laser would project upward onto the wall, the laser was mounted at a standardized 45° upward angle. The front of the force plate was placed 75 cm away from the wall. To capture the laser movement on the wall, the camera was placed directly in front of the force plate using a small 5 cm tall floor mount. The camera was mounted at a 45° upward angle to match the same angle of the laser. The placement of the force plate and camera was strategically chosen to allow for the entire cross-line laser projection to be captured by the camera.

Participants were then instructed to try their best to maintain a standardized testing position which was comprised of standing on only their involved limb, placing their hands on their hips, holding their contralateral limb in 30° of hip flexion and 45° of knee flexion, and positioning their eyes forward and not looking at the cross-line laser (Figure 1B) (34–37). While the participant maintained the balance position, the investigator simultaneously recorded data from the force plate and camera to capture measures of CoP and movement of the cross-line laser by manually starting both devices. The two instruments (force plate and camera) were not started from the same trigger; therefore, they were not perfectly synchronized. The force plate data collection software automatically stopped recording the trial after 10 s. The investigator manually stopped the camera recording via the remote after the force plate stopped recording. Between trials, participants were allowed to rest until they felt ready for the next trial.

Aligned with previous studies (14, 19), a trial was considered successful if the participant demonstrated the ability to maintain their hands on their hips, did not touch their contralateral foot to the ground or to the stance limb, and did not move the position of the stance limb foot. Once three successful trials of each condition were recorded or 10 attempts of the eyes-closed condition were performed, the testing session was complete. If the participant could not complete three successful trials in the eyes-open condition, they were excluded from the study. However, if they could complete three successful trials in the eyes-open condition, but not the eyes-closed condition, they were still included in the eyes-open data analysis.

### Data processing

Force plate data outcomes were processed and calculated using the associated software. Specific metrics utilized from the force plate included CoP average velocity and 95% area outcomes for each trial in both conditions. The average of the three successful trials in each condition (eyes-open and eyes-closed) was calculated for analysis.

For the cross-line laser processing, video files capturing the cross-line laser movement were uploaded to the tracking software and trimmed to 10 s (600 data points) to account for the camera being stopped after the fore plate. A virtual marker placed in the center of the cross-line for each trial was used to track the laser's movement. Each trial was played from start to finish with the virtual marker to determine if there were any instances when the virtual marker moved from the center of the cross-line laser so that it could be reassigned to the center if necessary. Data were then extracted from Kinovea based on the movement of the virtual marker (i.e., the cross-line laser). Data outcomes exported from Kinovea/movement of the cross-line laser included total distance, horizontal velocity (h-velocity), speed, and vertical velocity (v-velocity). A resultant velocity (r-velocity) was calculated using the Pythagorean theorem (i.e., h-velocity^2^ + v-velocity^2^ = r-velocity^2^). The average of the three successful trials (trials that matched the force plate recordings) of each outcome in both conditions (eyes-open and eyes-closed) was calculated for analysis.

### Statistical analysis

Using SPSS (IBM SPSS, V28), a Shapiro–Wilk test was conducted to calculate the distribution of all outcomes utilized in the study. Next, Pearson's correlation coefficients were calculated to determine the relationship between each cross-line laser and force plate outcome measures (CoP 95% area and CoP average velocity) in each condition (eyes-open and eyes-closed). The significance level was set *a priori* at *P* < 0.05 for all analyses. Correlations were interpreted as strong (*r* ≥ 0.7), moderate (0.7 > *r* ≥ 0.4), or weak (0.4 > *r* > 0) (38). For all variables that demonstrated a significant correlation, a simple linear regression was performed to determine the predictive value of the force plate outcomes from the laser outcomes. Lastly, a hierarchical linear regression determined if multiple cross-line laser outcomes together could more strongly predict the force plate outcomes with subsequent evaluation of collinearity for the variables in each model.

A second set of Pearson's coefficients and subsequent linear regressions determined the relationship of the cross-line laser outcomes in each condition with the participant's perceived stability of their ankle (i.e., number of episodes of giving way and IdFAI score) and perceived function of their ankle (i.e., FAAM-ADL and Sport scores).

## Results

All 24 participants were able to complete three trials of eyes-open single-limb balance; however, 4 participants were not included in the eyes-closed analysis due to insufficient successful trials in this condition. Participant demographics are reported in Table 1. All data were normally distributed (*W* = 0.93–0.97, *p* *=* 0.14–0.67).

**Table 1:** Participant demographics from this study.

Participant demographics	
Age	21.0 ± 3.0
Height (cm)	170.0 ± 8.6
Mass (kg)	75.4 ± 14.8
IPAQ	6,641.2 ± 5,795.2
IdFAI	22.6 ± 4.7
FAAM-ADL (%)	77.5 ± 13.0
FAAM-SPORT (%)	64.7 ± 16.7
Number of sprains	3.5 ± 1.6
Time since first sprain (months)	80.8 ± 54.3
Time since most recent sprain (months)	18.9 ± 29.1
Number of giving way episodes in the past 6 months	12.9 ± 14.6

### Eyes-open condition

Moderate-to-strong relationships (*r* = 0.46–0.87) were observed between cross-line laser measures (v-velocity, total distance, r-velocity, speed, and h-velocity) and force plate measures (CoP 95% area and CoP average velocity) (Table 2).

**Table 2:** Eyes-open and eyes-closed condition force plate outcomes center of pressure (CoP) 95% area and average velocity correlation (*r* = Pearson’s coefficient) and regression (*F*, *R*^2^), and hierarchical linear regression analysis to cross-line laser outcomes total distance, speed, horizontal velocity (h-velocity), vertical velocity (v-velocity), and resultant velocity (r-velocity).

Force plate outcome variables	Cross-line laser outcome variables	Pearson’s correlation	Regression model		Hierarchical regression models									
					Model 1		Model 2		Model 3		Model 4		Model 5	
		*r*	*F*	*R* ^2^	Δ*F*	*R* ^2^	Δ*F*	*R* ^2^	Δ *F*	*R* ^2^	Δ*F*	*R* ^2^	Δ*F*	*R* ^2^
Eyes-open condition														
CoP 95% area	Total distance	0.75**	27.5	0.56**	27.5	0.56**								
	Speed	0.75**	27.6	0.56**			0.06	0.56						
	h-velocity	0.82**	46.1	0.68**					8.44	0.69*				
	v-velocity	0.46**	6.0	0.21							0.04	0.69		
	r-velocity	0.74**	26.3	0.55**									2.22	0.72
CoP average velocity	Total distance	0.80**	39.7	0.64**	39.7	0.64**								
	Speed	0.80**	38.7	0.64**			0.05	0.64						
	h-velocity	0.87**	71.1	0.76**					10.42	0.77*				
	v-velocity	0.49*	7.0	0.24							0.13	0.77		
	r-velocity	0.78**	34.3	0.61**									5.9	0.83*
Eyes-closed condition														
CoP 95% area	Total distance	0.51*	6.4	0.26*	6.4	0.26*								
	Speed	0.61*	10.4	0.37*			10.02	0.54*						
	h-velocity	0.59*	9.8	0.35*					1.2	0.57				
	v-velocity	0.25	–	–							–	–		
	r-velocity	0.56*	8.1	0.31*									1.1	0.60
CoP average velocity	Total distance	0.56*	8.4	0.32*	8.4	0.32*								
	Speed	0.66*	14.0	0.44**			13.4	0.62*						
	h-velocity	0.62*	11.2	0.38*					0.5	0.63				
	v-velocity	0.31	–	–							–	–		
	r-velocity	0.60*	10.0	0.36*									2.2	0.68

*r* and *R*^2^ values: **p < 0.001, *p < 0.05.

Cross-line laser outcomes total distance, speed, h-velocity, v-velocity, and r-velocity predicted up to 68% of the variance in CoP 95% area (*R*^2^ = 0.21–0.68) (Table 2). Similarly, total distance, speed, h-velocity, v-velocity, and r-velocity predicted up to 76% (*R*^2^ = 0.24–0.76) of the variance in CoP average velocity (Table 2).

Total distance accounted for 55.6% of the variance in CoP 95% area in the first model of the hierarchical linear regression (Table 2). Inclusion of speed, v-velocity, and r-velocity did not significantly improve the model. In the final model, total distance and h-velocity were significant predictors, together explaining 69% (Table 2).

Regarding CoP average velocity, total distance accounted for 64.0% of the variance in the first model of the hierarchical linear regression (Table 2). Including speed and v-velocity did not significantly improve the model. In the final step of the model, adding h-velocity and r-velocity increased the explained variance to 83.0% (Table 2). Thus, total distance, h-velocity, and r-velocity together were significant predictors of CoP average velocity during eyes-open balance (Table 2).

**Table 3:** General ankle sprain history questions, ankle difficulty scores during activity (FAAM-sport % score), and ankle instability (IdFAI score) correlation (*r* = Pearson’s coefficient) and regression analysis (*F*, *R*^2^) to eyes-closed balance cross-line laser outcomes total distance, speed, horizontal velocity (h-velocity), vertical velocity (v-velocity), and resultant velocity (r-velocity).

Measures of participant perceived ankle stability	Cross-line laser outcome variables	Pearson’s correlation	Regression model	
		*r*	*F*	*R* ^2^
# of episodes of giving way in the past 6 months	Total distance	0.37	–	–
	Speed	0.45*	4.7	0.21*
	H-velocity	0.45*	4.6	0.20*
	V-velocity	0.16	–	–
	*R*-velocity	0.44	–	–
FAAM-Sport	Total distance	−0.36	–	–
	Speed	−0.44*	4.4	0.20*
	H-velocity	−0.42	–	–
	V-velocity	−0.33	–	–
	*R*-velocity	−0.43	–	–
IdFAI	Total distance	0.46*	4.9	0.21*
	Speed	0.50*	6.0	0.25*
	H-velocity	0.52*	6.8	0.27*
	V-velocity	0.19	–	–
	*R*-velocity	0.50*	6.0	0.25*

*r* and *R*^2^ values: *p < 0.05.

High collinearity values (tolerance: 0.002–0.12) in both hierarchical linear regressions indicated that only one of the cross-line laser variables (i.e., total distance or h-velocity) is needed to predict force plate outcomes.

No statistically significant relationships were found between eyes-open condition cross-line laser movement outcomes and the patient history questions FAAM-ADL, FAAM-SPORT, and IdFAI.

### Eyes-closed condition

Moderate relationships (*r*: 0.51–0.66) were observed between cross-line laser measures (total distance, r-velocity, h-velocity, and speed) and force plate measures (CoP 95% area and CoP average velocity) (Table 2).

Cross-line laser outcomes total distance, speed, h-velocity, and r-velocity predicted up to 37% of the variance (*R*^2^ = 0.26–0.37) (Table 2) of force plate CoP 95% area. Total distance, speed, h-velocity, and r-velocity predicted up to 44% of the variance in CoP average velocity (*R*^2^ = 0.32–0.44) (Table 2).

Total distance accounted for 26% of the variance in CoP 95% area in the first step of the hierarchical linear regression model (Table 2). Adding speed significantly improved this model, increasing explained variance to 54% (Table 2). With the addition of h-velocity and r-velocity in the final step, there was no significant change, indicating that total distance and speed were the best predictors of CoP 95% area (Table 2).

Regarding CoP average velocity, total distance explained 32% of the variance in the first step of the hierarchical linear regression model (Table 2). In the second step of the model, 62% of the variance was explained when adding speed (Table 2). The addition of h-velocity and r-velocity did not significantly improve the model (Table 2).

Collinearity values were high between the cross-line laser variables in each of the hierarchical linear regressions (tolerance: 0.04), indicating that only one of these cross-line laser variables is needed to predict force plate outcomes (i.e., total distance OR speed).

Moderate relationships were observed between the number of episodes of giving way in the past 6 months reported by the patient and cross-line laser outcomes, speed, and h-velocity (Table 3). Total distance, speed, h-velocity, and r-velocity were moderately to strongly correlated with IdFAI questionnaire scores (Table 3). Additionally, speed showed a moderate negative relationship with FAAM-Sport (Table 3).

Cross-line laser outcome speed and h-velocity predicted up to 21% (*R*^2^ = 0.20–0.21) of the variance in the number of episodes a participant reported in the previous 6 months (Table 3). Speed predicted 20.0% (*R*^2^ = 0.20) of the variance in FAAM-Sport score (Table 3). For IdFAI scores, total distance, speed, h-velocity, and r-velocity predicted up to 27% of the variance (*R*^2^ = 0.21–0.27) (Table 3).

## Discussion

The objective of this study was to validate the cross-line laser against the gold-standard force plate measures as a balance assessment tool for participants with CAI. The overall findings of this study suggest that most cross-line laser movement variables were predictive (*R*^2^ = 0.26–0.76) of traditional force plate measures during both eyes-open and eyes-closed balance. Thus, measures that were highly predictive of gold-standard measures (*R*^2^ > 0.70) may serve as a surrogate for a force plate when assessing balance among individuals with CAI.

Specifically, participants in this study who had a higher CoP 95% area and increased CoP average velocity on the force plate also had higher cross-line laser movement measures of total distance, speed, and horizontal and resultant velocities in both the eyes-open and eyes-closed conditions. Our results show that the cross-line laser movements had moderate-to-strong relationships to force plate outcomes and can predict anywhere from 22% to 76% of the variance in the force plate while participants balanced in an eyes-closed and eyes-open condition. While additional low-cost, clinically applicable balance assessments, including the BESS (17, 20, 21), SEBT (20), and foot-lift test (17, 21) exist, the cross-line laser may be a clinically friendly objective measurement tool for balance assessment. Specifically, the cross-line laser's ability to predict such a large variance in the force plate measures suggests it can detect subtle balance changes specific to the foot and ankle that previous clinical balance assessments may not capture.

Additionally, in our study, we explored the utility of multiple cross-line laser outcomes to force plate outcomes. However, our results indicate that only one variable of cross-line laser movement within each balance condition is needed to predict force plate outcomes. Specifically, we found that the horizontal velocity variable was the most predictive of force plate outcomes in the eyes-open condition, while speed was the most predictive variable in the eyes-closed condition. However, horizontal velocity was still highly predictive during the eyes-closed condition. Meaning that, across both balance conditions, the faster the cross-line laser moved horizontally, the larger the CoP area and average velocity captured by the force plate. In a clinical context, this indicates that only one cross-line laser variable, horizontal velocity, could be used to assess balance. This notion further adds to the simplicity of the cross-line laser for measuring balance.

Further supporting the use of the cross-line laser in individuals with CAI is the moderate relationships (*r* = 0.44–0.52) observed between some cross-line laser variables with patient-reported symptoms of CAI. Specifically, individuals who reported higher IdFAI scores and higher episodes of giving way also had higher cross-line laser movement measures of total distance, speed, and horizontal velocity during eyes-closed single-limb balance. Thus, these cross-line laser outcomes were able to capture some aspects of participant-perceived ankle instability and ankle function. Although a shared variance of 20%–27% can be considered small in some contexts, when factoring in the numerous impairments associated with CAI aside from balance deficits (39), being able to capture approximately 25% of the variance of perceived instability and self-reported function via the cross-line laser is quite remarkable. Stated differently, balance deficits are not the sole cause and contributor to the development of CAI (39); thus, we would not anticipate a balance tool to have 100% shared variance with symptoms of the condition. Although the cross-line laser tool should not replace validated questionnaires for quantifying perceived ankle instability and ankle function, the observed relationship and predictive nature do reinforce the importance of assessing and treating balance among individuals with CAI.

There are a few limitations to the implications of this study. First, during data processing, there were instances where the cross-line laser was moving too fast and causing the virtual marker to be dropped from the center of the laser output. When this occurred, a new marker was attached to the center of the laser output, and the tracking continued. These instances did not result in lost data. However, this did impede the automatic nature of the tracking process. This situation was observed primarily during eye-closed balance. Second, the force plate and camera for video recording could not be started synchronously. Therefore, some of the movement of the laser may have been lost during these moments potentially contributing to some of the non-statistically significant correlation and regression relationships. Had synchronization of the force plate and the cross-line laser occurred, we believe the relationship between the two outcomes would be stronger. Third, we did not have a healthy comparison population. Therefore, the cross-line laser cannot currently be used to determine the presence or absence of CAI.

Future studies could further validate the use of the cross-line laser among other patient populations with known balance impairments (i.e., knee and hip joint pathology, and concussion). Furthermore, the inclusion of a healthy population that is free from injury/illness in such studies could help determine the ability of the cross-line laser to discriminate between injury/illness status. Finally, establishing the ability of the cross-line laser to detect changes in balance over time will be important to tracking recovery from the corresponding injury/illness.

## Conclusion

Based on the results of this study, the cross-line laser is a valid means of balance assessment in individuals with CAI. Additionally, the cross-line laser is a capable tool for capturing some aspects of a patient's perceived ankle stability. The collective results of this study suggest that the cross-line laser is a valid tool to assess balance in individuals with CAI and that clinicians should consider utilizing given its low cost, accessibility, and ease of implementation.

## Data Availability

The raw data supporting the conclusions of this article will be made available by the authors, without undue reservation.
